# New multiplex PCR methods for rapid screening of genetically modified organisms in foods

**DOI:** 10.3389/fmicb.2015.00757

**Published:** 2015-07-24

**Authors:** Nelly Datukishvili, Tamara Kutateladze, Inga Gabriadze, Kakha Bitskinashvili, Boris Vishnepolsky

**Affiliations:** ^1^Ivane Beritashvili Center of Experimental BiomedicineTbilisi, Georgia; ^2^Faculty of Natural Sciences and Engineering, Ilia State UniversityTbilisi, Georgia

**Keywords:** genetically modified organism, food, screening, multiplex PCR, DNA marker

## Abstract

We present novel multiplex PCR methods for rapid and reliable screening of genetically modified organisms (GMOs). New designed PCR primers targeting four frequently used GMO specific sequences permitted identification of new DNA markers, in particular 141 bp fragment of cauliflower mosaic virus (CaMV) 35S promoter, 224 bp fragment of *Agrobacterium tumefaciens* nopaline synthase (NOS) terminator, 256 bp fragment of 5-enolppyruvylshikimate-phosphate synthase (*epsps*) gene and 258 bp fragment of Cry1Ab delta-endotoxin (*cry1Ab*) gene for GMO screening. The certified reference materials containing Roundup Ready soybean (RRS) and maize MON 810 were applied for the development and optimization of uniplex and multiplex PCR systems. Evaluation of amplification products by agarose gel electrophoresis using negative and positive controls confirmed high specificity and sensitivity at 0.1% GMO for both RRS and MON 810. The fourplex PCR was developed and optimized that allows simultaneous detection of three common transgenic elements, such as: CaMV 35S promoter, NOS terminator, *epsps* gene together with soybean-specific lectin gene. The triplex PCR developed enables simultaneous identification of transgenic elements, such as: 35S promoter and *cry1Ab* gene together with maize zein gene. The analysis of different processed foods demonstrated that multiplex PCR methods developed in this study are useful for accurate and fast screening of GM food products.

## Introduction

Modern biotechnology has revolutionized agro-food industry by introducing genetically modified organisms (GMOs). A large number of transgenic crops with new beneficial traits have been developed by insertion of foreign microbial genes into the plant genomes. The most common GM plants are herbicide-tolerant and insect-resistant soybeans, maize, cotton, and rapeseed. In recent years, both planting area and varieties of GM crops have been continuously increased (James, 2014). Global spread of genetically modified plants largely enhanced the share of GMOs in the worldwide distributed and commercialized plants, seeds, grains, food, and feed. Besides important benefits GM plants may pose both known and unknown risks to human health and the environment. Potential human health impacts mainly include allergens and toxins, transfer of antibiotic resistance markers. Potential environmental impacts include unintended transfer of transgenes through cross-pollination, unknown effects on other organisms (e.g., soil microbes), and loss of flora and fauna biodiversity (Sateesh, 2008). Correspondingly, experts and consumers have ambiguous opinions on GMOs. Increasingly, consumers are interested in GMO monitoring and labeling. To ensure the consumer's choice of freedom, many countries have established regulatory system and labeling regulation for genetically modified food (European Commission, 2003a,b).

The monitoring and traceability requests accurate and efficient methods for detecting GMOs in raw materials as well as in highly processed food. At present, numerous methods have been developed, validated, and harmonized worldwide for GMO detection (Dong et al., 2008; Michelini et al., 2008; Joint Research Centre, 2011; Zhang and Guo, 2011; Holst-Jensen et al., 2012; Broeders et al., 2014). DNA diagnostics represents the most efficient tool for GM food analysis as DNA is the most stable molecule during food processing. The most widely used reference methods for GMO detection rely on the different types of polymerase chain reaction (PCR) using primers specific for promoters, terminators, or inserted transgenes (Anklam et al., 2002; Marmiroli et al., 2008; Barbau-Piednoir et al., 2010, 2012; Joint Research Centre, 2011; Broeders et al., 2012; Milavec et al., 2014).

The traditional analytical procedure includes qualitative detection (screening), identification, and quantification of GMOs. Qualitative detection is the first critical step in GMO analysis, because only positive samples revealed during screening procedure are subjected to the further identification and quantitative analysis. At present, 357 GM events in 27 crop species have been approved for use as food, feed, or for environmental release (James, 2014). The screening for presence of GMO is expensive and time consuming due to the large number of GMO varieties on the market. Multiplex PCR approach is a promising mean to discriminate a growing number of transgenic events. It allows simultaneous amplification of several targets in a single reaction and ensures screening of agro-food products in a fast, cheap, and reliable manner. Multiplex PCR techniques have been applied for qualitative detection and identification as well as for quantification of GMOs (Hohne et al., 2002; Kim et al., 2006; Nikolić et al., 2008; Waiblinger et al., 2008; Samson et al., 2013). A number of new approaches (Querci et al., 2010; Pla et al., 2012) have been developed that involve the use of PCR with multiple targets and consecutive detection and identification of the amplification products using capillary gel electrophoresis (CGE) (Heide et al., 2008; Nadal et al., 2009; Holck et al., 2010), hybridization in microarrays (Leimanis et al., 2006; Hamels et al., 2009; Li et al., 2015), and next generation sequencing (Holst-Jensen et al., 2012; Milavec et al., 2014). The cost-efficiency and high-throughput could also be achieved by the combinatory SYBR Green qPCR and matrix-based approach (Chaouachi et al., 2008; Holst-Jensen, 2009; Querci et al., 2009; Van den Bulcke et al., 2010; Barbau-Piednoir et al., 2014).

Development of GMO detection methods is mainly focused on the GM soybean and maize as they are the most distributed transgenic crops worldwide. A number of reports described qualitative multiplex PCR methods for identification different lines of GM maize or GM soybean using event-specific primers (Hernandez et al., 2003; James et al., 2003; Germini et al., 2004; Onishi et al., 2005; Kim et al., 2013). While approach for identification of specific transgenic events is useful, it excludes the detection of other GM lines. Several studies described more beneficial strategy of multiplex PCR methods using primer pairs targeting commonly used regulatory elements and specific inserted genes as a rapid and convenient assay for the screening of GMOs. Matsuoka et al. (2000) described a method whereby primer pairs for the detection of several frequently used foreign DNA segments (including transgenes, promoter, and terminator regions) were designed and used in single-locus PCR. James et al. (2003) developed multiplex PCR procedure for detection of GM soybean. They used three GMO-specific primer pairs directed toward the *cp4-epsps* transgene, 35S promoter and NOS terminator together with two soybean-specific primer pairs targeting lectin and β–actin genes. The procedure developed was capable of reliably distinguishing non-GM soybean from RR soybeans and was successfully used to detect processed foods with Roundup Ready soybean (RRS). Forte et al. (2005) developed a molecular screening method based on multiplex-PCR that involves amplification of specific soya or maize sequences from plant DNA and the amplification of 35S promoter and NOS terminator for the detection of genetically modified soya and maize. Nikolić et al. (2008) developed screening methods based on triplex PCR that involve amplification of specific soybean and maize DNA sequences (lectin and zein genes) and the amplification of 35S promoter and NOS terminator for the detection of genetically modified soybean and maize. The multiplex PCR system has been described to amplify specific maize sequences from plant DNA (IVR and ZSSIIb), GMO genetic control elements 35S promoter, NOS terminator and Bt11 (IVS2/PAT) gene for the detection of GM maize (Zhang et al., 2010). Duplex PCR targeting *epsps* and *cry1Ab* genes allowed identification of GM materials in maize and soy samples simultaneously (Yoke-Kqueen et al., 2011).

Despite the developed and commercialized different techniques for GMO detection, there are a lot of gaps in this area; there are still unresolved issues in the development of appropriate testing methods, including: sampling strategies, extraction methods, multiplexing, quality assurance, economic impact of testing (Marmiroli et al., 2008). Therefore, there are growing demands for fast, cheap, and reliable methods to meet challenges for detection numerous plant species and hundreds of GMOs.

The objectives of this study were to develop cost-effective and time-saving methods for screening of GMOs and to determine the suitability of these methods for food analysis. Here, we propose four new DNA markers targeting important GMO specific sequences, such as cauliflower mosaic virus (CaMV) 35S promoter, NOS terminator, *epsps* gene, and *cry1Ab* gene. In addition, new multiplex PCR methods allowing simultaneous detection of the herbicide tolerance GM trait (CP4-EPSPS for Roundup Ready), CaMV 35S promoter, NOS terminator and soybean specific lectin gene as well as simultaneous detection of the insect control GM trait (Cry1Ab), CaMV 35S promoter and maize specific zein gene are presented and discussed. The application of these methods to GM reference materials as well as different food products is described and explained.

## Materials and methods

### Plant and food materials

Certified reference material (ERM-BF-410) of GM Soya bean powder set containing 0, 0.1, 0.5, and 10% Roundup Ready and maize GMO Standard (ERM-BF-413) set for 0, 0.1, 0.5, 1, 2, and 5% MON 810 were purchased commercially (Fluka, Biochemika). The commercially available food products, namely soya flakes, soybean sauce, crispbread, dried crust, bread 1, bread 2, maize flour, flakes 1, flakes 2, and chips as well as seeds of soybean (*Glycine max*) and maize *(Zea mays)* were obtained from local markets. The electric grinder was utilized for the milling of dried foods and seeds to obtain flours. Some foodstuffs such as soy sauce, maize chips and flakes were frozen in liquid nitrogen and ground using a mortar and pestle.

### DNA extraction

The genomic DNA was extracted from 100 mg material by two methods, such as DNeasy plant mini kit (Qiagen) or cetyltrimethyl ammonium bromide (CTAB)-based method. The method was chosen foreseen our previous studies (Kutateladze et al., 2009; Datukishvili et al., 2010). In particular, CTAB-based method was used for foodstuffs while DNeasy plant mini kit was utilized for powders. The DNA cleanliness and concentration were estimated by spectrophotometer (Genova Life Science Analyzer, Jenway).

### Bioinformatic analysis and design of oligonucleotide primers

The PCR primers used in this study are shown in Table 1. The species-specific primer pairs targeting soybean lectin gene and maize zein gene were taken from our previous publications (Kutateladze et al., 2013; Gabriadze et al., 2014). The published GenBank databases and DNA sequences were screened for the identification of GMO-specific DNA sequences and PCR primers. We selected four commonly used transgenic sequences such as CaMV 35S promoter, *Agrobacterium tumefaciens* NOS terminator, 5-enolppyruvylshikimate-phosphate synthase (*epsps*) gene and Cry1Ab delta-endotoxin (*cry1Ab*) gene and designed different primer pairs targeting to them using online resources. Firstly, the PCR primers were chosen by Primer-BLAST (Ye et al., 2012) and PrimerQuest tool (https://eu.idtdna.com/PrimerQuest). In addition, the sequence alignment tool Align_MTX (Vishnepolsky and Pirtskhalava, 2009) was used for final design, however possible formation of dimers and secondary structures was evaluated by FastPCR (Kalendar et al., 2011). The fitness of the primer pairs for multiplex PCR system was checked using MultiPLX (Kaplinski et al., 2005) and FastPCR. Oligonucleotide primers were got from Eurofins MWG Operon.

**Table 1:** **Oligonucleotide primers used in PCR**.

**Target**	**Primers**	**Sequences**	**Amplicon (bp)**	**References**
Soybean lectin gene	LECT for/LECT rev	ACGGCACCCCAAAACCCTCG GGAAGCGGCGAAGCTGGCAA	101	Kutateladze et al., 2013
Maize zein gene	ZEIN for/ZEIN rev	ACACCACCGACCATGGCAGC TGGTGGCAAGTGCGCTGGAA	102	Gabriadze et al., 2014
CaMV 35S promoter	P35S for/P35S rev	CGTGCACCATGATGTGTGATTCGAC GTGGGATTGTGCGTCATCCCTT	141	This study
NOS terminator	TNOS for/TNOS rev	GGTACCGGATCCAATTCCCGATCGTT CGCGCTATATTTTGTTTTCTATCGCGT	224	This study
EPSPS gene	EPSPS for/EPSPS rev	ACCGGCCTCATCCTGACGCT CCGAGAGGCGGTCGCTTTCC	256	This study
Cry1Ab gene	Cry1 for/Cry1 rev	GCACCTCCGTGGTGAAGGGC AACCCACGGTGCGGAAGCTG	258	This study

### PCR analysis

The thermal cycler Techne TC-412 was used for all PCR analysis. The uniplex and multiplex PCR conditions were optimized by testing of different parameters, such as primer concentration (0.1–1.0 μM), MgCl_2_ concentration (1.5–5 mM), number of cycles (25–50), annealing temperature (50–67°C), and elongation time (20–60 s).

The reagents from New England BioLabs were applied for the uniplex PCR analysis. The amplifications were performed in a final volume of 25 μl containing the following: 60–70 ng of genomic DNA, 1.5 mM MgCl_2_, 0.2 mM of each dNTP (Deoxynucleotide solution mix), standard Taq Buffer with 1.25 units of Taq DNA polymerase, and 0.5 μM of each primer. The Qiagen multiplex PCR master mix consisting of 1.25 units HotStarTaq DNA polymerase was used for the multiplex PCR analysis. In addition, the reaction volume of 25 μl contained 90 ng of genomic DNA, 3 mM MgCl_2_, and the following primer concentrations: 0.3 μM LECTf/LECTr; 0.2 μM P35Sf/P35Sr, 0.5 μM TNOSf/TNOSr, 0.3 μM EPSPSf/EPSPSr in fourplex PCR (**Figure 2**) while 0.3 μM ZEINf/ZEINr, 0.2 μM P35Sf/P35Sr, 0.25 Cry1f/Cry1r in triplex PCR.

The uniplex PCRs with primer pairs P35Sf/P35Sr, TNOSf/TNOSr, and EPSPSf/EPSPSr had the same optimal cycling profile, such as preincubation at 95°C for 3 min, 40 cycles consisting of DNA denaturation at 95°C for 30 s, primer annealing at 65°C for 30 s, elongation at 72°C for 35 s; final extension step at 72°C for 5 min. The PCR cycling conditions for primer pair Cry1f/Cry1r were as follows: denaturing at 95°C for 3 min, 50 cycles of 25 s at 95°C, 30 s at 62°C, 45 s at 72°C; final extension at 72°C for 7 min. The multiplex PCR conditions were as follows: preincubation for 5 min at 95°C, 40 cycles of 30 s at 95°C, 30 s at 65°C, 30 s at 72°C; final extension step for 7 min at 72°C. The amplification products were analyzed by electrophoresis in 2.0% agarose gels containing 1 μg/ml of Ethidium Bromide (EtBr).

## Results and discussion

### Identification of GMO-specific DNA markers

GMO screening methods are mainly targeted to the regulatory elements and genes for specific traits presented in GMOs. To ensure detection of the most GMOs four frequently used GMO specific sequences were selected, in particular CaMV 35S promoter (P35S), *Agrobacterium tumefaciens* NOS terminator (TNOS), 5-enolppyruvylshikimate-phosphate synthase (*epsps*) gene and Cry1Ab delta-endotoxin (*cry1Ab*) gene. A large number of transgenic plants contain the CaMV 35S promoter and/or NOS terminator control elements (James et al., 2003; Barbau-Piednoir et al., 2010; Querci et al., 2010). The Roundup Ready (CP4- EPSPS-based glyphosate herbicide tolerance) and the insect resistance (Cry1Ab Bt) traits are present in a high number of different GMOs (Barbau-Piednoir et al., 2012). The herbicide tolerant GM crops contain the enzyme 5-enolpyruvylshikimate-3-phosphate synthase (*epsps*) gene, isolated from *Agrobacterium tumefaciens* strain CP4. The insect resistant GM crops contain the gene of insecticidal protein Cry1Ab δ-endotoxin from *Bacillus thuringiensis* ssp. kurstaki.

**Figure 1:**
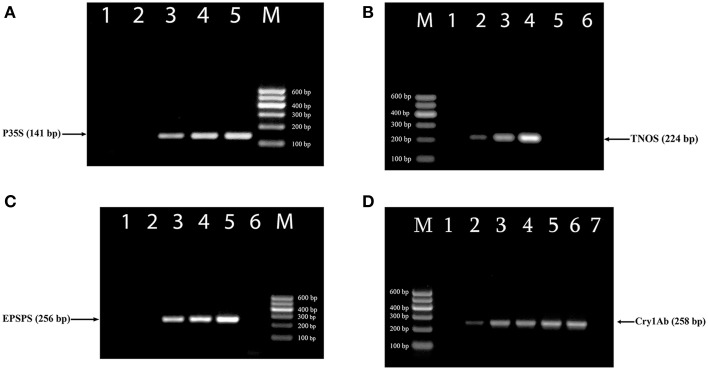
**Uniplex PCR amplification of GMO-specific regions using primer pairs: P35S for/P35S rev (A), TNOS for/TNOS rev (B), EPSPS for/EPSPS rev (C), Cry1 for/Cry1 rev (D)**. Samples **(A)**: lane 1, water, lanes 2–5. Roundup Ready soybean (RRS set): 0, 0.1, 0.5, and 10%; **(B)** lane 1–4. RRS set: 0, 0.1, 0.5, and 10%; lane 5. soybean seeds, lane 6. water; **(C)** lane 1, soybean seeds, lanes 2–5. RRS set: 0, 0.1, 0.5, and 10%; lane 6. water; **(D)**. lanes 1–6. Maize MON 810 set: 0, 0.1, 0.5, 1, 2, and 5%, lane 7. water. **M**, Molecular weight marker (Qiagen GelPilot 100 bp ladder): 100, 200, 300, 400, 500, and 600 bp.

The efficiency of new designed primer pairs was separately tested to amplify their targets by uniplex PCRs (Figure 1). RRS was used as a target for three primer pairs complementary to the 35S promoter, NOS terminator and *epsps* gene (Figures 1A–C) as they exist in this transgenic soybean line as well as MON 810 GM maize line was template for *cry1Ab* relevant primer pairs (Figure 1D). Each uniplex PCR gave the expected one amplicon for GMO samples (Table 1). The P35Sfor and P35Srev primer pair directed toward the CaMV 35S promoter, amplified a 141 bp DNA fragment (Figure 1A); the TNOSfor and TNOSrev primer pair targeting the NOS terminator amplified a fragment of 224 bp (Figure 1B); the EPSPSfor and EPSPSrev primer pair directed to the *epsps* gene, produced a 256 bp PCR product (Figure 1C), and the Cry1for and Cry1rev primer pair targeting *cry1Ab* gene amplified a fragment of 258 bp (Figure 1D). Analysis of certified reference materials containing 0, 0.1, 0.5, and 10% RRS and 0, 0.1, 0.5, 1, 2, and 5% MON810 showed high sensitivity (at least 0.1% GM material) of these PCR methods. Moreover, the intensity of the DNA band increased correspondingly to the increased amount of transgenic material in the samples. No PCR fragment was observed for non-GM soybean seeds, 0% RRS, 0% MON810 and water control (Figure 1) indicating high specificity of the GMO detection methods. The GMO-specific PCR fragments identified in this study are distinguished from the DNA markers described previously (Dong et al., 2008; Joint Research Centre, 2011).

### Multiplex PCR for GMO detection

**Figure 2:**
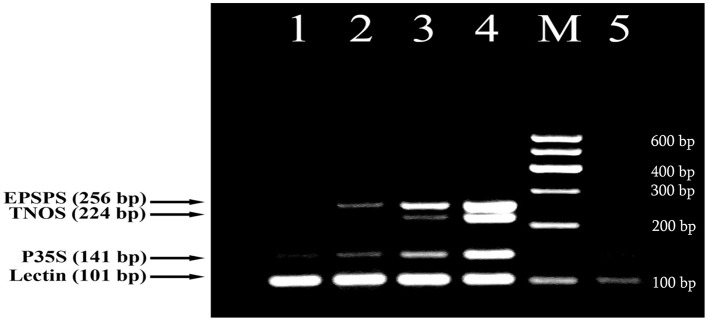
**Fourplex PCR for detection of GM Soybean (Roundup Ready soya) using primer pairs: LECT for/LECT rev, P35S for/ P35S rev, TNOS for/ TNOS rev, and EPSPS for/ EPSPS rev**. Lanes 1–4. Certified reference material of Roundup Ready soybean (RRS) set: 0%, 0.1% RRS, 0.5% RRS, 10% RRS, lane 5. water. **M**, Molecular weight markers (Qiagen GelPilot 100 bp ladder): 100, 200, 300, 400, 500, and 600 bp.

In this study, a fourplex PCR was designed and tested for effective detection of GM soybean. Four primer pairs were identified based on the developed DNA markers (Figure 1) together with soybean-specific amplicon (Kutateladze et al., 2013) to discriminate GM and non-GM conventional soybeans. The three GMO specific primer pairs such as P35Sfor/P35Srev (141 bp product), TNOSfor/TNOSrev (224 bp product) and EPSPSfor/EPSPSrev (256 bp product) were used for identification of transgenic regions of RRS. The other primer pair LECTfor/LECTrev (101 bp product) was applied for detection of endogenous lectin gene to check the amplifiability of soybean DNA. The analysis of GM Soya bean powder set containing 0, 0.1, 0.5, and 10% Roundup Ready allowed to check sensitivity and reliability of the PCR method. The 101 bp PCR product existed in all soybean samples including blank (0% RRS) sample, as was expected. The expected amplification products of 141, 224, and 256 bp were present in all transgenic samples (Figure 2) except of 224 bp DNA band corresponding to the NOS terminator in the 0.1% RRS sample. The 0.1% GMO sample generated the weak bands, whereas the samples containing 0.5 and 10% RRS produced clearly visible bands. Moreover, the intensity of the DNA bands increased correspondingly to the increased amount of transgenic material in the samples. No PCR product was observed in negative (non-DNA) sample. The results obtained indicate that the developed multiplex PCR method enables to detect GMO with a high specificity and sensitivity at least (0.1% GMO). Moreover, this procedure can be used for screening a broad range of GMOs, including non-approved varieties if they contain one of the amplicons identified by this method.

**Figure 3:**
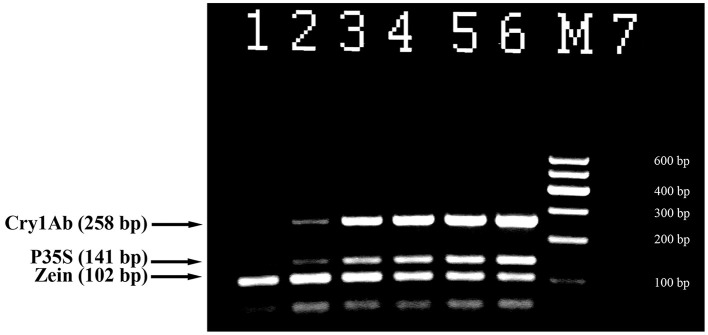
**Triplex PCR for detection of MON 810 maize using primer pairs: ZEIN for/ZEIN rev, P35S for/P35S rev, and Cry1 for/ Cry1 rev**. Lanes 1–6. Certified reference material of MON810 maize set: 0% (blank), 0.1, 0.5, 1, 2, and 5%, lane 7. water. **M**, Molecular weight markers (Qiagen GelPilot 100 bp ladder): 100, 200, 300, 400, 500, and 600 bp.

Triplex PCR was developed for the effective detection of genetically modified maize. The primer pair ZEIN for and ZEIN rev (102 bp product) targeting endogenous zein gene was used to check amplifiable maize DNA; primer pairs P35S for and P35S rev (141 bp product), Cry1 for and Cry1 rev (258 bp product) were utilized for detection of GM maize (Figure 3). All maize samples including non-GM maize generated the expected PCR fragment of 102 bp size and equal intensity (Figure 3). The analysis of certified reference materials containing MON810 maize set ranging from 0.1 to 5% GM content demonstrated high sensitivity and accuracy of the assay. The primer pairs P35S for and P35S rev as well as Cry1 for and Cry1 rev gave the expected amplicons of 141 and 258 bp in size when GM maize (including 0.1% GMO) DNA was applied as a template (Figure 3, lanes 2–6). The intensity of the amplification products was increased correspondingly to the increasing percentage of GM content in the samples. However, no PCR products were observed for non-GM maize and negative control (Figure 3, lanes 1, 7) that confirmed specificity of the assay.

### Application to foods

**Figure 4:**
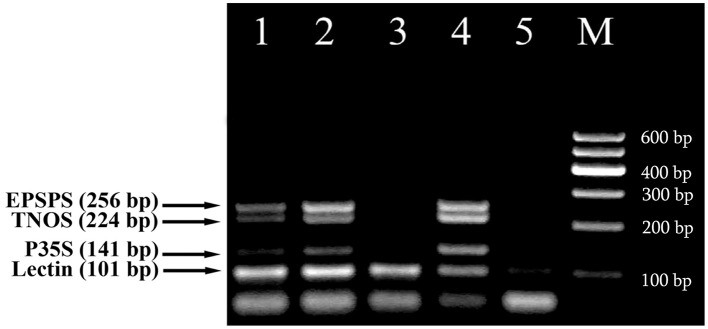
**Screening of different soybean products to evaluate the presence of soybean and GMOs using fourplex PCR with primer pairs: LECT for/LECT rev, P35S for/P35S rev, TNOS for/TNOS rev, and EPSPS for/EPSPS rev**. Lanes 1, 2. Certified reference material of Roundup Ready soybean (RRS): 0.1% RRS and 0.5% RRS, lane 3. soybean seeds, lane 4. Soya flakes, lane 5. Soybean sauce, **M**, Molecular weight markers (Qiagen GelPilot 100 bp ladder): 100, 200, 300, 400, 500, and 600 bp.

These methods were then used for testing of foods to evaluate the eventual matrix effect. Different commercially available soybean food products, such as seeds, flakes and sauce as well as wheat products, namely two kinds of bread, dried crust and crispbread were analyzed with the fourplex PCR using soybean specific primers LECT for/LECT rev and three GMO-specific primers, namely P35S for/P35S rev, TNOS for/TNOS rev, and EPSPS for/EPSPS rev. The 101 bp PCR product was seen in all soy food samples while soya sauce gave weak signal due to the small amount of the extracted genomic DNA (Figure 4). RRS was found to be present in soybean flakes. They generated three PCR fragments with the sizes of 141, 224, and 256 bp corresponding to the CaMV 35S promoter, NOS terminator and *epsps* gene, respectively (Figure 4, lane 4). In addition, side by side PCR analysis of flakes sample with unknown GM content with identified GMO standards, such as 0.1% RRS and 0.5% RRS (Figure 4, lanes 1, 2), allowed comparative evaluation of GM content in flakes that is more than 0.5% RRS.

**Figure 5:**
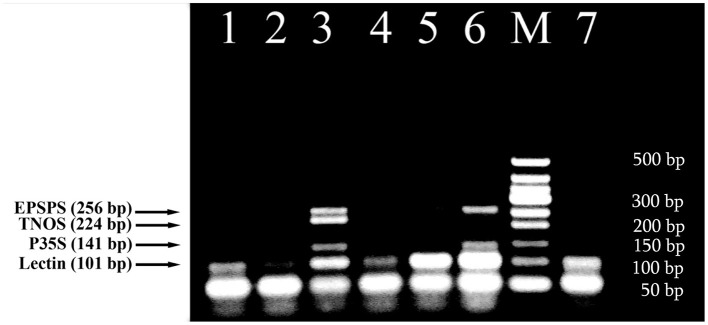
**Screening of different wheat products to evaluate the presence of soybean ingredients and GMOs using fourplex PCR with primer pairs: LECT for/LECT rev, P35S for/P35S rev, TNOS for/TNOS rev, and EPSPS for/EPSPS rev**. Lane 1. bread 1, lane 2. Bread 2, lane 3. 0.5% RRS, lane 4. crispbread, lane 5. 0% RRS, lane 6. dried crust, 7. soybean seeds. **M**, Molecular weight markers (Qiagen GelPilot 50 bp ladder): 50, 100, 150, 200, 250, 300, 350, 400, and 500 bp.

The intriguing results were obtained from multiplex amplification of wheat products. As seen in Figure 5, soybean appears to exist with different concentrations in three samples 1, 4 and 6. In addition, comparative analysis of wheat products with reference standards of RRS clearly exhibited presence of GM soybean with concentration about 0.5% GMO in the sample of dried crust (Figure 5, lanes 3, 6).

**Figure 6:**
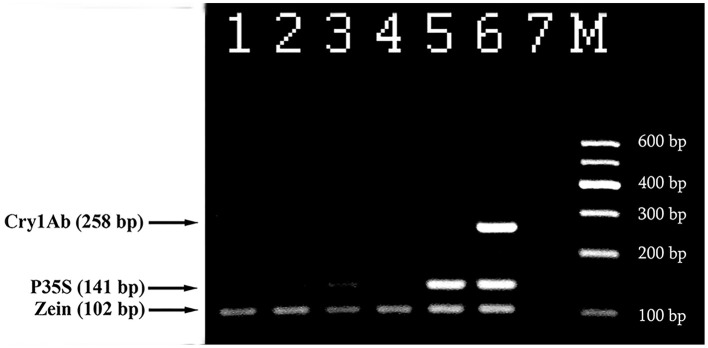
**Screening of different maize products to evaluate the presence of maize and GMOs using threeplex PCR with primer pairs: ZEIN for/ZEIN rev, P35S for/P35S rev and Cry1 for/Cry1 rev**. Lane 1. flour, lane 2. chips, lane 3. Flakes 1, lane 4. Flakes 2, lane 5. 2% maize Bt176, lane 6. 2% maize MON810, lane 7. Water. **M**, Molecular weight markers (Qiagen GelPilot 100 bp ladder): 100, 200, 300, 400, 500, and 600 bp.

Figure 6 represents analysis of maize foodstuffs and two varieties of genetically modified maize, such as Bt176 and MON810 by triplex PCR described above. Maize MON810 generated three amplified products of sizes of 102, 141, and 258 bp (Figure 6, lane 6) as was expected, however Bt176 produced only two 102 and 141 bp PCR amplicons (Figure 6, lane 5). Maize Bt176 did not give 258 bp amplicon corresponding to the Cry1A(b) δ-endotoxin gene despite of the fact that this gene is inserted in the transgenic Bt176 variety. The comparative sequence analysis of the transgenic regions of the MON810 and Bt176 allowed explain the obtained result. It appears that primers Cry1for and Cry1rev have exact complementary sequences to the *cry1Ab* gene of maize MON810 but they are quite different from the suitable regions of the *cry1Ab* gene in Maize Bt176 (Table 2). As a whole, nucleotide sequences of *cry1Ab* gene from MON810 and Bt176 differ by approximately 13%. Our outcomes confirm data about different variants of the *cry1Ab* gene introduced into various GM maize varieties (Barbau-Piednoir et al., 2012). The consequences suggested that triplex PCR method described in this study may be used for identification of MON810 by three amplicons and Bt176 by two amplicons. One hundred and two base pairs PCR product corresponding to endogenous zein gene was clearly seen in all maize samples as was expected, however water control did not produce any signals indicating high specificity and reliability of the multiplex assay (Figure 6). In addition, one very weak DNA band of 141 bp was amplified by flakes 1 (Figure 6, lane 3), that suggested about existence of low content of Bt176 in this product. Figure 6 demonstrates the advantage of this assay over other screening methods as it may be used to distinguish two GM maize varieties, namely MON810 and Bt176 besides GMO detection.

**Table 2:** **Sequence alignment between regions corresponding to the primers Cry1for and Cry1rev in the maize MON810 and Bt176**.

**Sequences**	**Primers**
GCACC**TC**CGTGGTGAAGGGC	Cry1for
GCACC**TC**CGTGGTGAAGGGC	MON810
GCACC**AG**CGTGGTGAAGGGC	BT176
A**AC**C**CA**CG**G**TG**C**G**G**A**A**G**C**TG	Cry1rev
A**AC**C**CA**CG**G**TG**C**G**G**A**A**G**C**TG	MON810
A**CA**C**GG**CG**C**TG**G**G**C**A**G**G**T**TG	BT176

*The mismatched nucleotides are highlighted in bold*.

## Conclusion

Multiplex PCR procedures that facilitate rapid and reliable screening of GMOs in foods were developed. The application of these methods reduces the costs and time of analysis as they detect two major GM traits, the Cry1A and the CP4-EPSPS and two generic recombinant markers, the CaMV 35S promoter and the *Agrobacterium* NOS terminator, as the most frequently present targets in GMO to date (Barbau-Piednoir et al., 2012). The results obtained from testing of the food samples side-by-side with Fluka reference materials indicate that these PCR tests may be easily adapted for semiquantitative analysis of GM foods. The procedures of sample preparation and DNA purification described above (see Materials and Methods) facilitated and enhanced DNA extraction. The template and primer concentrations were critical factors in some reactions. The optimized conditions for uniplex and multiplex PCRs were more dependent on the primer combinations and concentrations than the DNA source. These findings coincide with the outcomes of James et al. (2003). The PCR systems developed for GM soybean and maize detection were also successfully used for analysis of different foodstuffs.

Analytical procedures described here allow reliable, efficient and fast screening of GMOs, namely Roundup Ready and Bt crops as well as other transgenic plants containing GMO-specific elements such as: 35S promoter, NOS terminator, *epsps* gene and *cry1Ab* gene. Correspondingly, the developed multiplex PCR methods may be implemented easy to control distribution and use of GM foods.

### Conflict of interest statement

The authors declare that the research was conducted in the absence of any commercial or financial relationships that could be construed as a potential conflict of interest.
